# Championing Second Careers and Online Education at an Age-Friendly UIndy

**DOI:** 10.1093/geroni/igaf122.1005

**Published:** 2025-12-31

**Authors:** Lisa Borrero, Marwa Noureldin

**Affiliations:** University of Indianapolis, Indianapolis, Indiana, United States; University of Indianapolis, Indianapolis, Indiana, United States

## Abstract

The University of Indianapolis’s (UIndy) path to becoming a member of the Age-Friendly University (AFU) Global Network reflects a strategic commitment to fostering a more inclusive educational environment for individuals across the lifespan. As part of the plan to prepare for AFU membership, UIndy established a campus-wide task force to assess the university’s strengths and areas of opportunity in promoting age-inclusive practices. Additionally, through a series of surveys targeting UIndy’s alumni and retired employees and focused listening sessions with current faculty and staff, the university collected valuable insights on engagement and opportunities for improvement. These findings helped shape the university’s approach to the AFU application, which emphasized principles #2 and #5 of the AFU framework—principles that align with UIndy’s new mission to champion lifelong learning and vision to provide educational opportunities “Anytime. Anywhere. For life.” UIndy’s commitment to these principles is further demonstrated through the development of the new UIndy Online and Sease Institute, which aims to expand access to online education, support second careers, and foster lifelong learning. This holistic approach underscores UIndy’s dedication to intergenerational connection and meeting the needs of an aging population while advancing its mission of inclusive, flexible, and lifelong educational opportunities.

